# Supplementary figures and images for: Plasma IL-6 levels following corticosteroid therapy as an indicator of ICU length of stay in critically ill COVID-19 patients
Source: Cell Death Discov. 2021 Mar 15;7:55. doi: 10.1038/s41420-021-00429-9 (PMC7958587; doi:10.1038/s41420-021-00429-9)

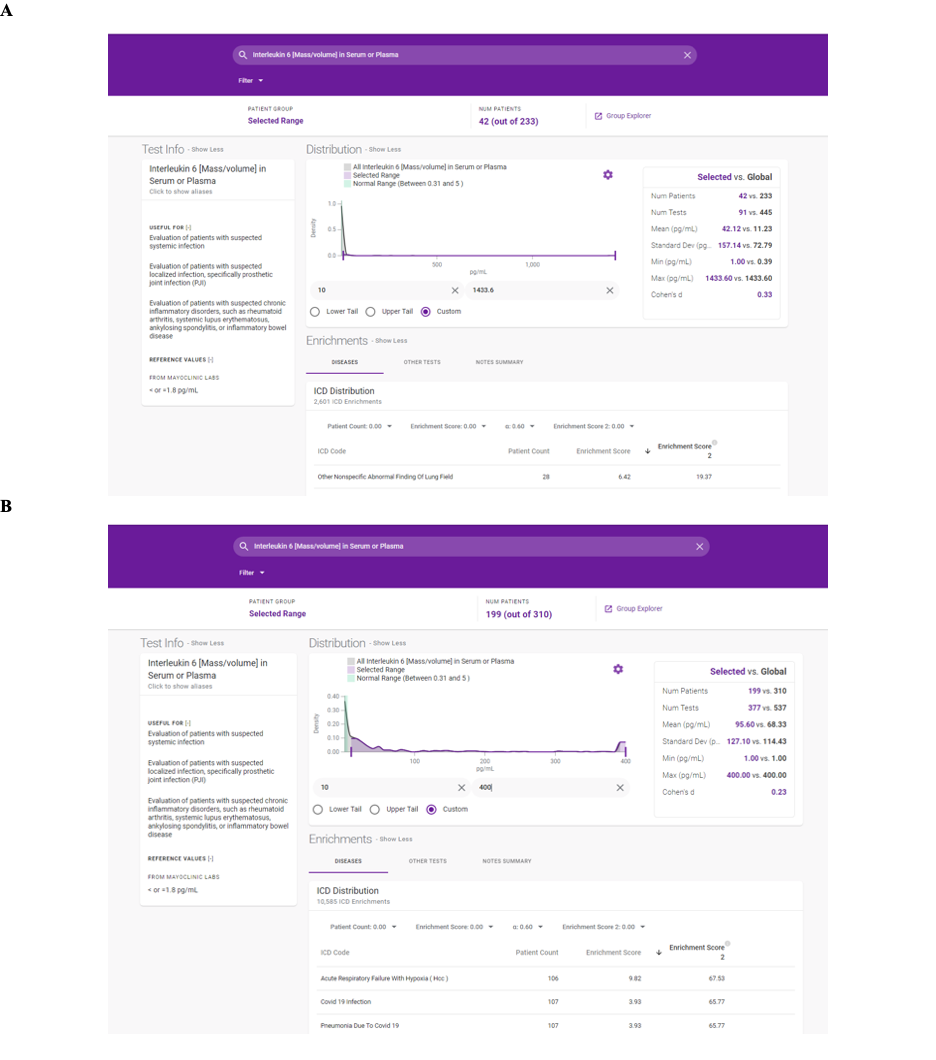

Supplement: Supplementary file 1 — Supplementary Figure 1.1 [file 41420_2021_429_MOESM1_ESM.png]

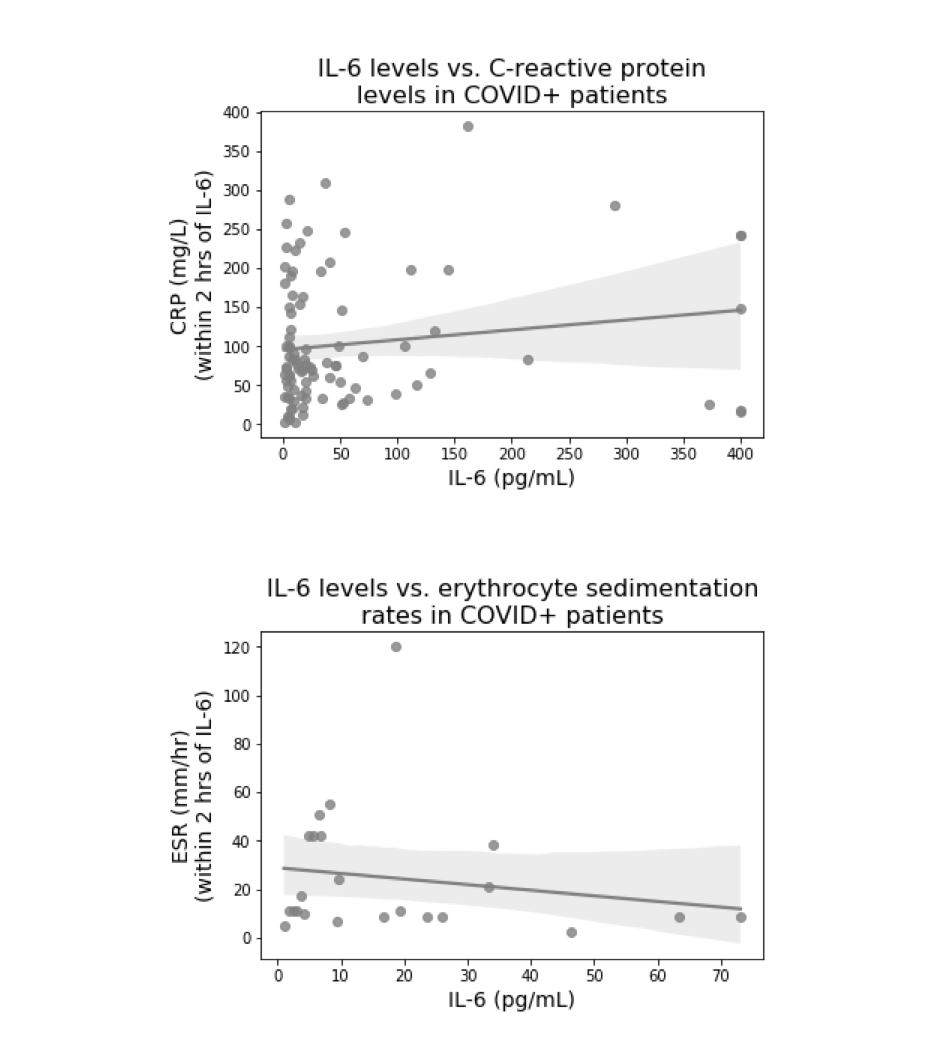

Supplement: Supplementary file 2 — Supplementary Figure 1.2 [file 41420_2021_429_MOESM2_ESM.png]

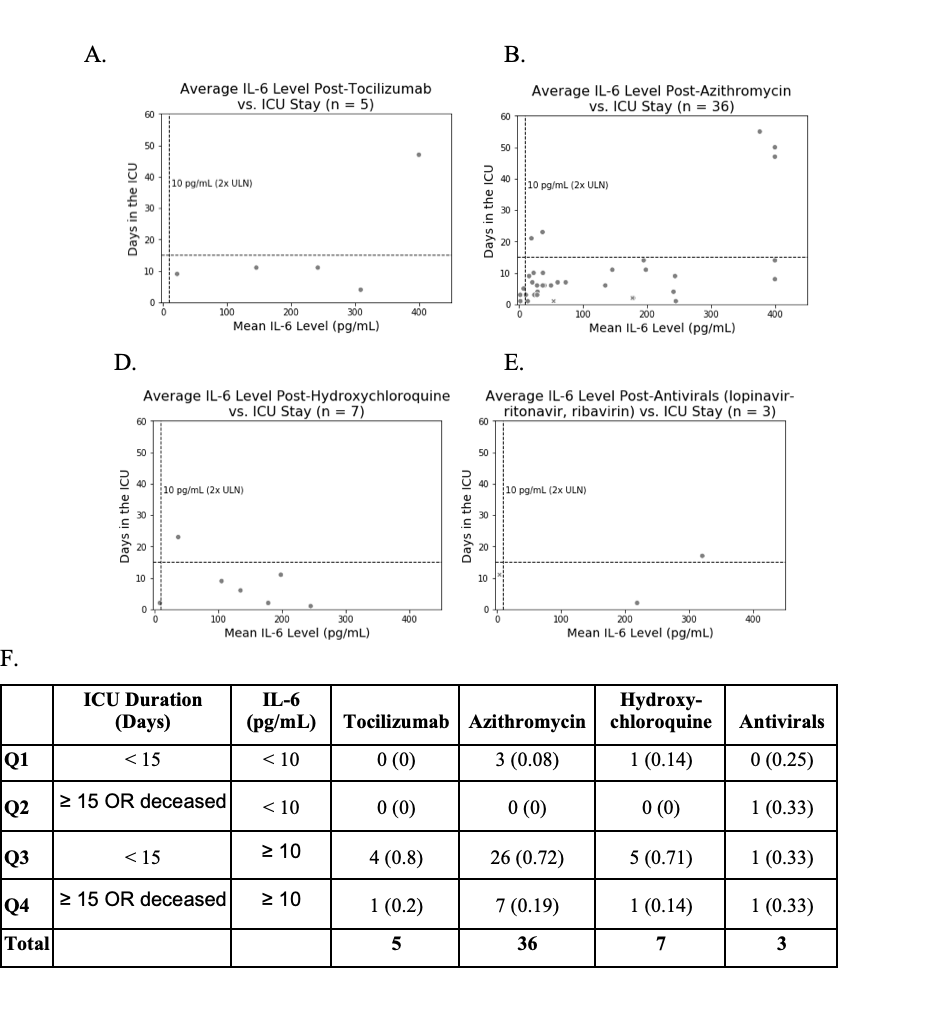

Supplement: Supplementary file 3 — Supplementary Figure 1.3 [file 41420_2021_429_MOESM3_ESM.png]

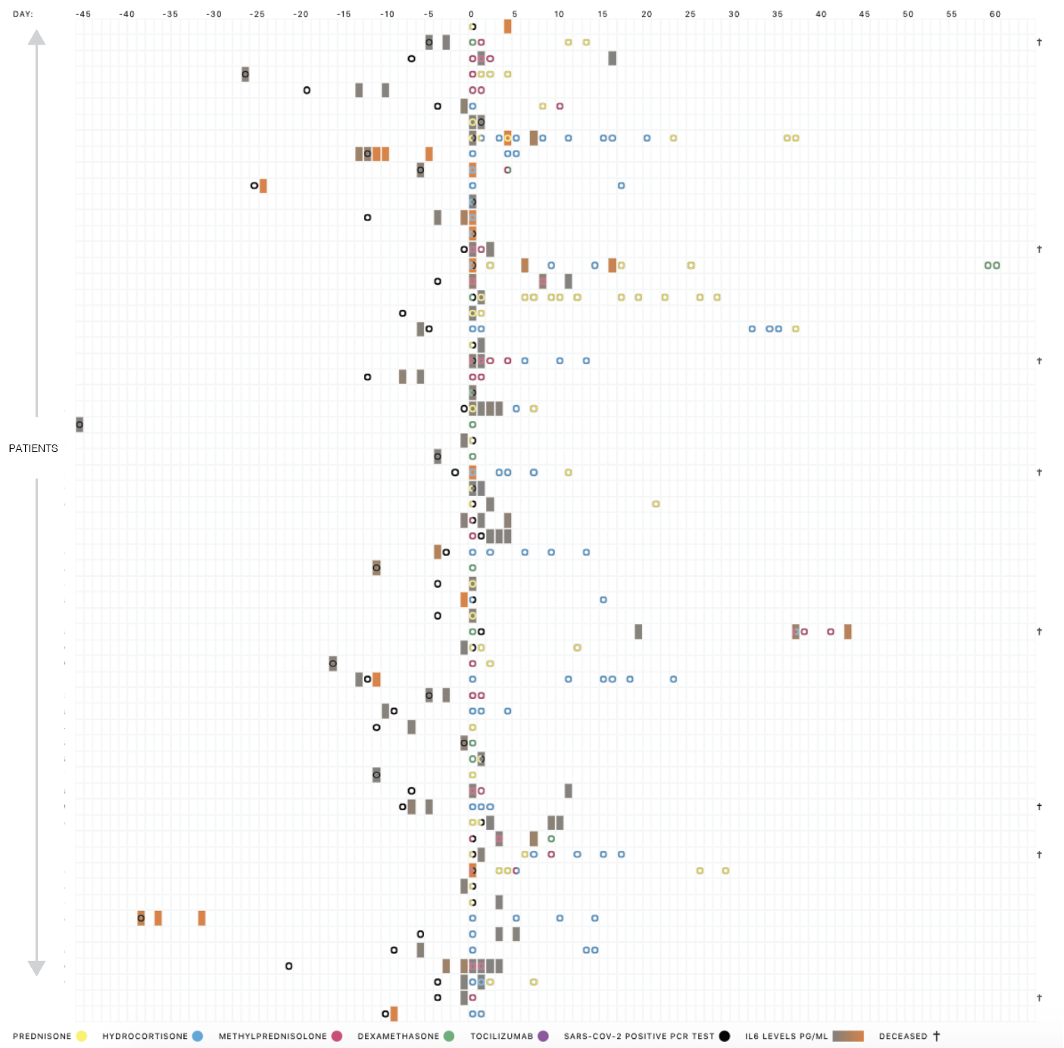

Supplement: Supplementary file 4 — Supplementary Figure 2 [file 41420_2021_429_MOESM4_ESM.png]

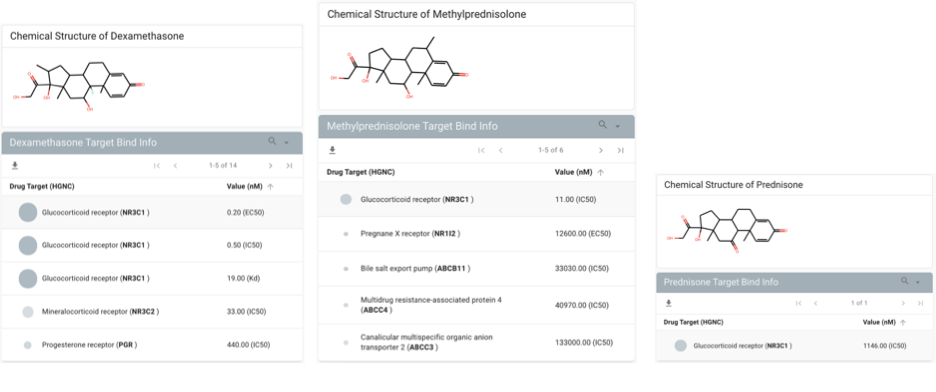

Supplement: Supplementary file 5 — Supplementary Figure 3.1 [file 41420_2021_429_MOESM5_ESM.png]

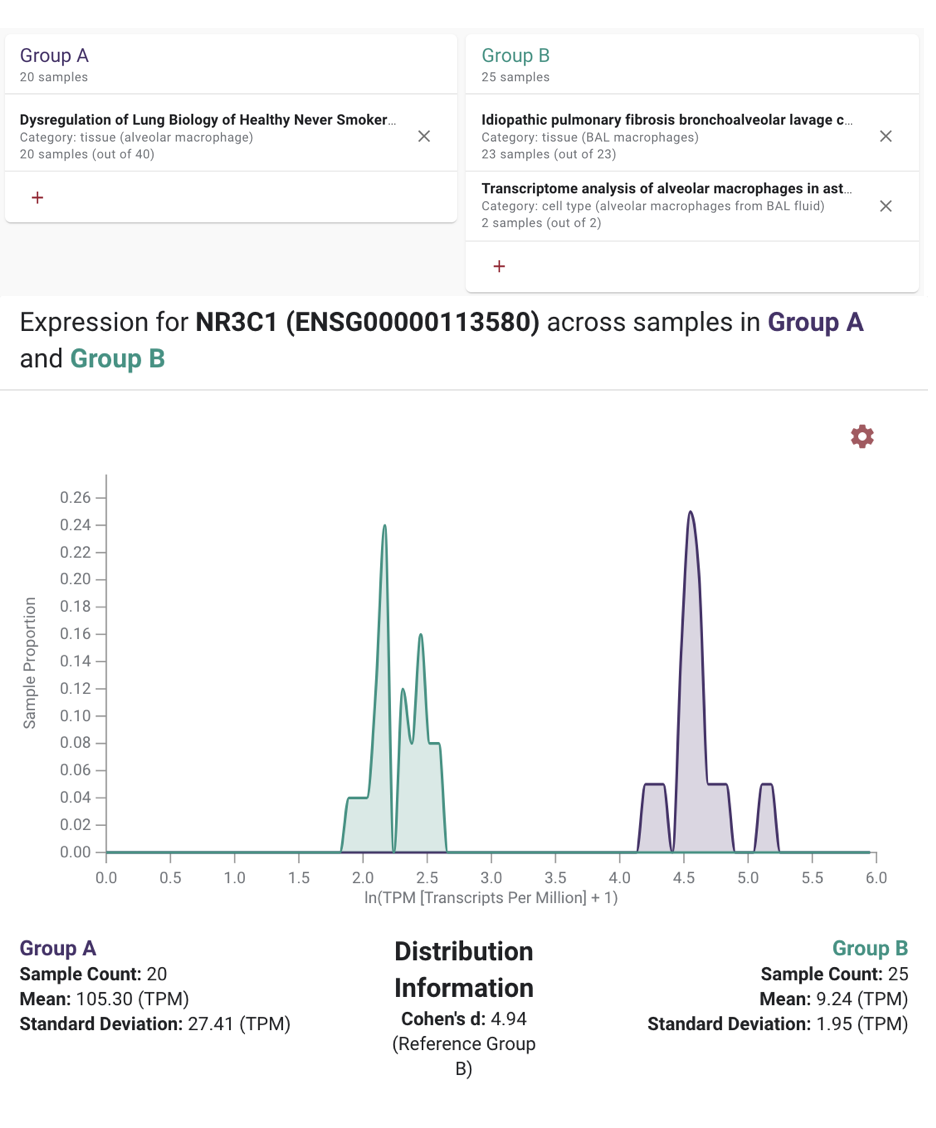

Supplement: Supplementary file 6 — Supplementary Figure 3.2 [file 41420_2021_429_MOESM6_ESM.png]

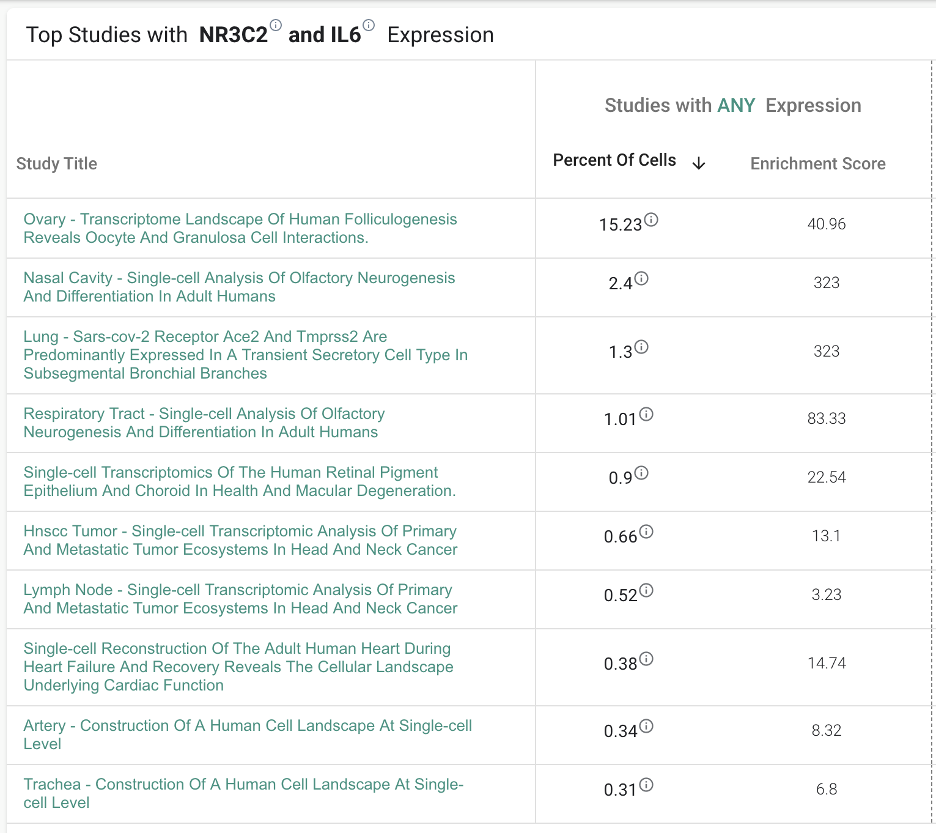

Supplement: Supplementary file 7 — Supplementary Figure 3.3 [file 41420_2021_429_MOESM7_ESM.png]
