# Supplementary material for: Plasma IL-6 levels following corticosteroid therapy as an indicator of ICU length of stay in critically ill COVID-19 patients
Source: Cell Death Discov. 2021 Mar 15;7:55. doi: 10.1038/s41420-021-00429-9 (PMC7958587; doi:10.1038/s41420-021-00429-9)
Supplement: Supplementary file 8 — Supplementary Figure Legends [file 41420_2021_429_MOESM8_ESM.docx]

**Supplementary Material**

**Figure 1 - Supplementary Figure 1.** Distribution of serum or plasma IL-6 measurements for a subset of 700k patients at the Mayo Clinic taken in 2019 **(A)** and 2020 **(B)**. Also shown are the enriched ICD codes for the patients in the right tail of the distribution with abnormally high IL-6 levels.

**Figure 1 - Supplementary Figure 2.** Relationship between the inflammatory markers C-reactive protein (CRP) and the erythrocyte sedimentation rate and IL-6 in hospitalized COVID-19 patients. In each case, blood for inflammatory marker measurement was collected within 2 hrs of IL-6 blood collection. The linear regression is shown with a dark grey line, and 95% confidence intervals around the regression are shown in light grey. In both cases, no significant relationship between IL-6 and levels of the inflammatory marker is found.

**Figure 1 - Supplementary Figure 3.** **Relationship between IL-6 levels and ICU duration for COVID-19 ICU patients who had at least one plasma IL-6 measurement after therapeutic administration.** Longitudinal measurements of plasma IL-6 in patients after they received (**A)** Non-topical corticosteroids (**B**) Tocilizumab (**C**) Azithromycin (**D**) Hydroxychloroquine (**E**) antivirals, at least once at or after the diagnosis of COVID-19 via SARS-CoV-2 PCR. (**F**) Patient counts and proportions for each quadrant, where deceased patients were counted with those in the ICU ≥ 15 days.

**Figure 2 - Supplementary Figure 1.** Characterization of the overall cohort of n = 63 COVID_pos_ patients in the ICU that received a SARS-CoV-2 positive PCR test (black), a therapeutic treatment (other colors) and plasma IL-6 lab test (gray-to-orange gradient).

**Figure 3 - Supplementary Figure 1.** Characterization of molecular targets of dexamethasone, methylprednisolone, and prednisone. NR3C1 is the highest affinity target for each of these corticosteroids.

**Figure 3 - Supplementary Figure 2.** Expression of NR3C1 by bulk RNA-sequencing in alveolar macrophages from three independent studies. In one study (Group A, purple), alveolar macrophages were found to express high levels of NR3C1, with 13 of 20 samples falling in the top 5% of NR3C1 expression considering all human samples deposited in GEO. In two other studies (Group B, green), alveolar macrophages expressed lower but still appreciable levels of NR3C1.

**Figure 3 - Supplementary Figure 3.** Single cell RNA-seq studies showing non-zero (any) co-expression of NR3C2 and IL-6. Primary oocytes, granulocytes, and secondary oocytes of the ovaries show significant co-expression of NR3C2 and IL-6. The other cell types only have minor populations of cells that co-express both NR3C2 and IL-6.
